# Fluctuations in dispensed out-patient psychotropic medication prescriptions during the COVID-19 pandemic in The Netherlands

**DOI:** 10.1192/bjo.2024.867

**Published:** 2025-03-20

**Authors:** Damian A. Visser, Daphne S. Everaerd, Hannah Ellerbroek, Janneke R. Zinkstok, Indira Tendolkar, Femke Atsma, Arnt F. A. Schellekens

**Affiliations:** Research Institute for Medical Innovation, Radboudumc, Nijmegen, The Netherlands; Nijmegen Institute for Scientist-Practitioners in Addiction, Nijmegen, The Netherlands; Donders Institute for Brain, Cognition and Behaviour, Nijmegen, The Netherlands; Karakter, Child and Adolescent Psychiatry, Nijmegen, The Netherlands; Radboud Institute for Health Sciences, Scientific Centre for Quality of Healthcare, Radboud University Medical Centre, Nijmegen, The Netherlands

**Keywords:** COVID-19 pandemic, lockdown, national registry data, prescriptions, psychotropic medication

## Abstract

**Background:**

The COVID-19 pandemic and lockdowns had a significant impact on mental well-being and (mental) healthcare systems globally.

**Aims:**

To describe trends and dynamics of out-patient prescribing of psychotropic medications during the COVID-19 pandemic in The Netherlands.

**Method:**

Dispensed psychotropic medication prescriptions during the COVID-19 pandemic from March 2020 to March 2022 were retrieved from national registry data. Numbers of total and incident dispensed prescriptions and defined daily doses (DDDs) were identified for six medication groups. Overall pandemic-related changes in prescribing trends were analysed using interrupted time-series analyses. Lockdown-related prescribing dynamics were described using monthly risk ratios.

**Results:**

No overall pandemic-related changes in prescribing were detected, except for alcohol addiction medication, for which a pre-pandemic decline in total dispensed prescriptions and DDDs levelled off during the pandemic: +10 prescriptions per week (95% CI 7–11, *P* ≤ 0.001) and +111 DDDs per week (95% CI 56–165, *P* = 0.001). Monthly prescribing dynamics showed transient increases in all medication groups during the second and third lockdown periods. There were decreases in dispensed incident antidepressant and opioid addiction medication prescriptions during the first lockdown (average risk ratios: 0.87 and 0.88 respectively), and DDDs of dispensed incident and total attention-deficit hyperactivity disorder medication prescriptions and incident benzodiazepine prescriptions were elevated from the end of the second lockdown (average risk ratios: 1.40, 1.12 and 1.17, respectively).

**Conclusions:**

These findings raise concerns regarding possible over- and under-prescribing during the pandemic. Further understanding of specific factors driving these changes is necessary to help prepare for future mental health(care) challenges.

The World Health Organization declared coronavirus disease 2019 (COVID-19) a global pandemic on 11 March 2020. Preventive measures against population spreading, also called ‘lockdowns’, led to social isolation, loneliness, psychological stress and decreased physical activity,^1^ which are well-known risk factors for the emergence and deterioration of mental disorders.^2^ Previous international studies on the effects of the COVID-19 pandemic and lockdown measures on population mental health have shown mixed results. For example, Shah and colleagues^3^ reported increased psychiatric symptoms in general populations, whereas Pan and colleagues^4^ found no such change. In Europe, an increase in psychiatric symptom prevalence rates at the beginning of the pandemic was found, paralleled by a decrease in the incidence of new psychiatric diagnoses; this could be indicative of reduced availability of psychiatric care.^5^

## Prescriptions as a proxy

As well as population prevalence and incidence studies, psychotropic medication prescription data can serve as a proxy for population mental health and for the availability and accessibility of psychiatric care during the COVID-19 pandemic.^6–13^ Throughout the pandemic in The Netherlands, (mental) healthcare visits were periodically either cancelled, delayed or converted to telephone or video appointments when possible, owing to (local) regulations or quarantining by patients. In turn, these adjustments may have influenced the prescription patterns of psychotropic medications. For example, relative over-prescribing could have been be driven by the lack of availability of psychotherapy, whereas relative under-prescribing could be caused by hesitancy of physicians to initiate pharmacotherapy via telemedicine.^6^ The pandemic seems to have affected prescription patterns in different countries inconsistently. For instance, in England, antidepressant prescriptions increased during the period of January to August 2020, peaking in March 2020,^12^ but they showed an overall decline between 2020 and 2022.^13^ However, prescriptions of anxiolytic and hypnotic medication in England increased above predictions based on pre-pandemic trends. Prescriptions of psychiatric medications, mainly antidepressants and benzodiazepines, showed increasing trends during the pandemic in France.^8^ However, a study of Polish prescription data demonstrated no significant change in prescription rates of antidepressants and benzodiazepines during the pandemic.^14^ A US study also found stable incident prescription patterns for antidepressants and benzodiazepines, whereas incident attention-deficit hyperactivity disorder (ADHD) medication prescriptions substantially increased.^6^

## Knowledge gaps

Previous studies of psychotropic medication prescription dispensing patterns during the COVID-19 pandemic have focused on a limited set of medication groups, mainly antidepressants and benzodiazepines. Therefore, changes in prescription patterns of other psychiatric medications, such as antipsychotics and addiction medications, remain relatively unexplored. Furthermore, most prescription-based research has analysed changes over the COVID-19 pandemic as a whole, regarding it as a homogenous stressor over time. Although this is valuable for discovering overall pandemic-related effects, more detailed time-dependent analyses could provide additional insight into potential (transient) lockdown-related prescribing dynamics. In this way, potential drivers of these changes could be more accurately identified and possibly mitigated in the future. Previous studies have also focused on either total or incident prescriptions, instead of considering both simultaneously. Finally, whereas many studies considered amounts of dispensed prescriptions, defined daily doses (DDDs) should also be taken into account to enable monitoring of total dispensed medication amounts in a population over time.

## Study aims

The comprehensive aim of this study was to describe relative changes in dispensed prescriptions of six psychotropic medication groups during 2 years of the COVID-19 pandemic in The Netherlands. This was achieved using the following two approaches.

- Overall pandemic-related changes in dispensed prescription trends were examined using interrupted time-series analyses (ITSAs). In these analyses, prescribing trends of a pre-pandemic reference period (March 2019 to March 2020) were compared with the overall trend during the COVID-19 pandemic (March 2020 to March 2022).
- Potential lockdown-related dispensed prescription dynamics were investigated using monthly risk ratio analyses to assess the risk of a person in the population receiving a psychotropic medication prescription in a COVID month compared with a corresponding pre-pandemic reference month.

By combining these approaches, fluctuations in psychiatric prescribing during the pandemic can be described in a more detailed manner, including detection of (periods of) possible over- and/or under-prescribing within psychotropic medication groups. A distinction was also made between the total numbers of dispensed prescriptions and incident prescriptions to provide insight in changes in treatment continuation and initiation during the pandemic, respectively. In addition to amounts of dispensed prescriptions, DDDs were included in the analyses to describe changes in dispensed medication amounts in the study population over time. Potential drivers of the discovered changes in prescribing trends and dynamics are discussed and contrasted with (inter)national findings.

## Method

### Study population and design

A national registry-based time series study was performed with out-patient prescription data from the national pharmacy database Genees- en hulpmiddelen Informatie Project, from the Dutch National Health Care Institute (Zorginstituut Nederland).^15^ A list of dispensed prescriptions was requested for medications with Anatomical Therapeutic Chemical (ATC) codes^16^ that had psychiatric conditions as their main indication according to the Dutch pharmacotherapy formulary.^17^ The medication groups included in this study did not have any group-specific regulations regarding maximum prescription sizes. Data were included from a period from 1 year before the COVID-19 pandemic (11 March 2019 to 8 March 2020, defined as the ‘reference year’) and for 2 years during the pandemic (9 March 2020 to 13 March 2022, defined as the ‘COVID period’).

Data were provided in aggregated form and stratified by ATC code, dispensing calendar week (with a total of 157 weeks), patient demographics (age and gender) and prescription type (incident or repeated prescriptions). An incident prescription was defined as a prescription dispensed to an individual who had not received a prescription with the same ATC code in the past year. Owing to the aggregated delivery of the data, prescription data were fully anonymised and therefore did not require ethics review.

### Data conversions

Individual ATC codes were combined to form the following six psychotropic medication groups based on the main indication: ADHD medication (*n* = 4), antipsychotics (*n* = 23), benzodiazepines (*n* = 17, including z-drugs), alcohol addiction medication (*n* = 4), opioid addiction medication (*n* = 3) and antidepressants (*n* = 22). Numbers of dispensed prescriptions and DDDs were aggregated in these medication groups for further analysis. See Supplementary Table 1 available at https://doi.org/10.1192/bjo.2024.867 for an overview of the ATC codes included in each medication group. Certain medications within the originally defined ‘lithium’ and ‘nicotine addiction’ groups underwent significant changes with respect to insurance coverage from January 2020. These groups were therefore excluded from the study before statistical analysis owing to an inability to meaningfully distinguish changes related to health insurance coverage from possible pandemic-related changes.

To correct for population growth, the numbers of dispensed prescriptions and DDDs were expressed as numbers of prescriptions or DDDs per 100 000 individuals in the Dutch population. For risk ratio analysis, data aggregated by week were further aggregated by month to compare more relevant time periods during the COVID-19 pandemic with the reference year (thus, month to month as opposed to week to week).

### Statistical analyses

**Fig. 1:**
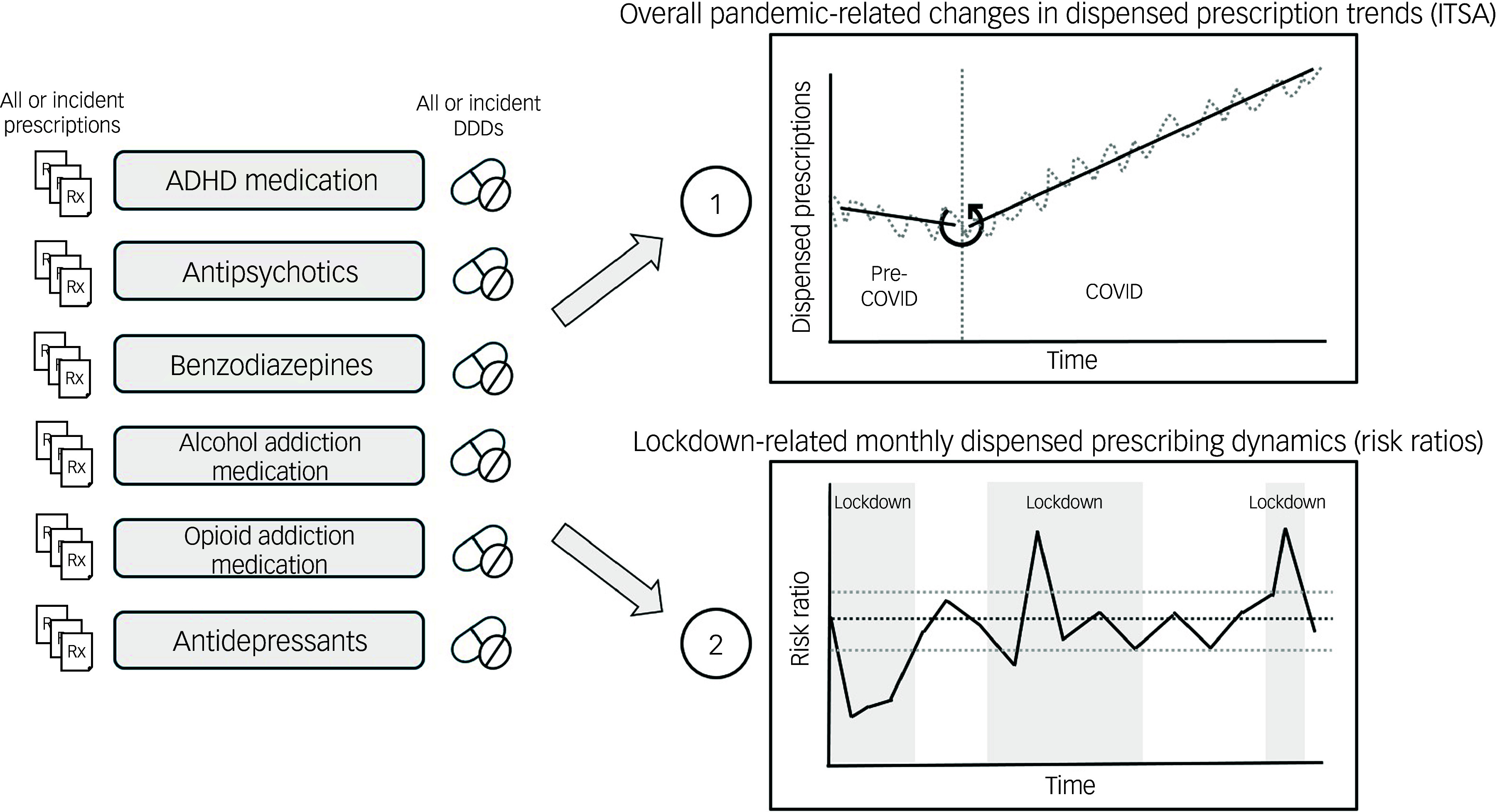
Schematic overview of the two main statistical analyses performed in the six medication groups of the study: (1) overall pandemic-related changes in dispensed prescription trends using interrupted time series analysis (ITSA); and (2) lockdown-related dispensed prescription dynamics with risk ratios. (Incident) prescriptions and defined daily doses (DDDs) were separately analysed. ADHD, attention-deficit hyperactivity disorder.

See Fig. 1 for a schematic overview of the two main statistical analyses performed in this study. To determine whether the COVID-19 pandemic had an overall impact on dispensed psychotropic prescription trends, an ITSA was performed per medication group, using an autoregressive integrated moving average (ARIMA) model. An ARIMA (1,0,0) model was used to adjust for first-order autocorrelations. The reference period contained 52 weeks: week 11 of 2019 to week 10 of 2020. The 53rd time point in the ARIMA model (week 11 of 2020) was defined as the start of the COVID-19 pandemic. The COVID period lasted from week 11 of 2020 to week 11 of 2022. The weekly number of dispensed prescriptions or DDDs of the reference year was used to model the counterfactual trend during the COVID-19 pandemic and tested for a change in slope from the 11th week of 2020 onwards. ITSAs were two-sided, with Bonferroni corrections applied to all *P*-values. Model adjustment for seasonality was not performed owing to a single reference year.

A monthly risk ratio was calculated for each medication group to view relative dispensed prescription rate dynamics and also ameliorate recurring seasonal prescribing fluctuations.^18,19^ The risk ratio was calculated using the following formula:

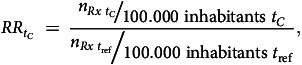

where *RR_tC_* is the risk ratio at a given month during the COVID pandemic (*t_C_*). *RR_tC_* was calculated by dividing the total number of dispensed prescriptions in a given COVID month (*n*_*Rx*_ *t_C_*) per 100 000 Dutch inhabitants by the number of prescriptions in the same reference month before the pandemic (*n*_*Rx*_ *t*_ref_) per 100 000 individuals. In the case of DDDs, *n*_*Rx*_ *t_C_* could be converted to number of DDDs (*n*_DDD_ *t_C_*). Changes in risk ratio of ±0.1 from the zero-change value of 1.0 were predefined as relevant for further discussion.^18^

### Definition of lockdown periods

In these analyses, lockdown periods were annotated in visualisations of the monthly risk ratio analyses over time. The Oxford COVID-19 Government Response Tracker (OxCGRT)^20^ was used to uniformly define lockdown periods in this study. The OxCGRT is a longitudinal global panel database of COVID-19 pandemic policies, which provides a numerically expressed aggregated score based on nine policy indicators including school closures and travel restrictions. Data were extracted from the database as daily values on a continuous scale from 0 (least strict response) to 100 (strictest response). Daily scores were converted to weekly scores, whereby the highest value within that particular week was selected. The weighted average stringency index for vaccinated and non-vaccinated individuals was used from 25 September 2021 onwards. Lockdown periods were defined as OxCGRT scores of ≥50 for ≥4 weeks to prevent extraneous division of the lockdown periods (Supplementary Figs 1 and 2).^18^

Regarding the OxCGRT cut-off score of 50, this value was first reached in The Netherlands when the first major national mitigation measures occurred (e.g. more widespread stay-at-home orders, cancellation of public events, closure of schools and child day care, closure of restaurants and bars, travel limitations, and initiation of the national crisis framework). The World Health Organization also declared the COVID-19 outbreak as a pandemic on 11 March 2020, corresponding with week 11 of 2020. Thus, the following three Dutch lockdown periods were defined and annotated in the monthly risk ratio figures: week 11 of 2020 to week 27 of 2020 (lockdown 1), week 34 of 2020 to week 25 of 2021 (lockdown 2) and week 47 of 2021 to week 4 of 2022 (lockdown 3). Sensitivity analyses were performed with an OxCGRT cut-off to 60: this had no impact on the overall findings or main conclusions. Data were analysed using IBM SPSS Statistics version 29.0.

## Results

### Sample characteristics

More than 59 million dispensed prescriptions and 1.4 billion DDDs were included in the total research period of 3 years (from March 2019 to March 2022). During the whole COVID-19 period, the total number of dispensed psychotropic prescriptions decreased by 2%, whereas DDDs of dispensed psychotropic medications increased by 3% compared with the reference year (Table 1). Four per cent of all the included prescriptions were incident prescriptions, and this was similar during the COVID and reference periods.

**Table 1:** Numbers of dispensed prescriptions and defined daily doses stratified by medication group, before and during the COVID-19 pandemic

Time period	Full study period	Reference year	COVID period
	Week 11 2019 to week 10 2022	Week 11 2019 to week 10 2020	Week 11 2020 to week 10 2022
Mean population size	17 460 557	17 366 741	17 496 926
Total dispensed Rx	59 024 988	19 909 506	40 184 367
Total dispensed DDD	1 483 387 148	482 742 474	1 021 813 377

Medication group^a^	Females, %	Mean age per Rx, years	Total Rx, *n* (%)	Incident Rx, %	Per 100 000 inhabitants per year		Per 100 000 inhabitants per year			
					Rx	DDD	Rx	DDD	Δ reference year, %	
									Rx	DDD
ADHD medication	36	28	4 634 161 (8)	5	8680	334 864	8953	363 572	+3	+9
Antipsychotics	49	53	12 939 076 (22)	3	24 909	248 308	24 667	253 852	−1	+2
Benzodiazepines	62	60	13 390 461 (23)	6	26 069	432 542	25 384	439 699	−3	+2
Opioid addiction	24	54	1 309 519 (2)	1	2516	44 280	2499	45 353	−1	+2
Alcohol addiction	35	51	287 612 (0)	9	595	11 427	528	10 077	−11	−12
Antidepressants	66	59	26 464 159 (45)	4	51 872	1 708 275	49 990	1 745 834	−4	+2
Total by Rx	58	55	59 024 988 (100)	4	114 641	2 779 696	112 019	2 858 386	−2	+3

ADHD, attention-deficit hyperactivity disorder; DDD, defined daily dose; Rx, prescriptions.

a. Data for the medication groups include total numbers of prescriptions and numbers of prescriptions per year per 100 000 inhabitants, as well as percentage incidence prescriptions, percentage female prescription receivers and average DDD per prescription per medication group. The mean age per medication group was calculated as the average age per prescription. Population data (mean population size) were obtained from Statistics Netherlands (cbs.nl).

### Overall pandemic-related changes in dispensed prescription trends

For overall pandemic-related changes in dispensed prescription trends, a significant change in alcohol addiction medication was detected with the ITSA (Table 2). This change is also shown in Supplementary Fig. 3. Total numbers of prescriptions and DDDs increased by +10 prescriptions (95% CI: 8–12, *P* ≤ 0.001) and +111 DDDs per week (95% CI: 56–165, *P* = 0.001), respectively, compared with the reference year. However, no significant change could be detected for dispensed incident prescriptions for alcohol addiction medication or for any other medication groups during the pandemic.

**Table 2:** Overall pandemic-related prescribing trend changes using interrupted time-series analysis with autoregressive integrated moving average modelling: slope change for weekly numbers of dispensed (total and incident) prescriptions and defined daily doses

Drug groups	Number of Rx						Defined daily dose					
	Total Rx			Incident Rx			Total Rx			Incident Rx		
	Slope change	95% CI	*P*-value^a^	Slope change	95% CI	*P*-value	Slope change	95% CI	*P*-value^a^	Slope change	95% CI	*P*-value
ADHD medication	7.13	−84.45 to 98.70	1.000	−4.11	−15.76 to 7.55	1.000	646.20	−2889.38 to 4181.79	1.000	−119.38	−451.66 to 212.90	1.000
Antipsychotics	28.37	−49.42 to 106.15	1.000	−2.35	−11.41 to 6.71	1.000	66.71	−989.34 to 1122.76	1.000	21.96	−37.52 to 81.45	1.000
Benzodiazepines	8.97	−66.09 to 84.04	1.000	0.02	−12.60 to 12.64	1.000	330.74	−1178.98 to 1840.47	1.000	167.60	−31.13 to 366.33	0.587
Opioid addiction	11.86	−3.08 to 20.65	0.051	0.25	−0.11 to 0.60	1.000	115.41	−43.05 to 115.41	0.914	1.43	−2.42 to 5.27	1.000
Alcohol addiction	9.58	7.54 to 11.61	<0.001*	0.61	0.05 to 1.18	0.206	110.60	55.74 to 165.45	0.001*	9.95	−6.73 to 26.63	1.000
Antidepressants	108.28	−189.09 to 190.18	1.000	11.09	−11.99 to 34.16	1.000	2130.23	−6890.99 to 11151.45	1.000	320.40	−244.01 to 884.82	1.000

ADHD, attention-deficit hyperactivity disorder; Rx, prescriptions.

a. Asterisk indicates results significant at 95% confidence level with Bonferroni corrections applied.

### Lockdown-related risk ratio dynamics for monthly dispensed prescriptions

**Fig. 2:**
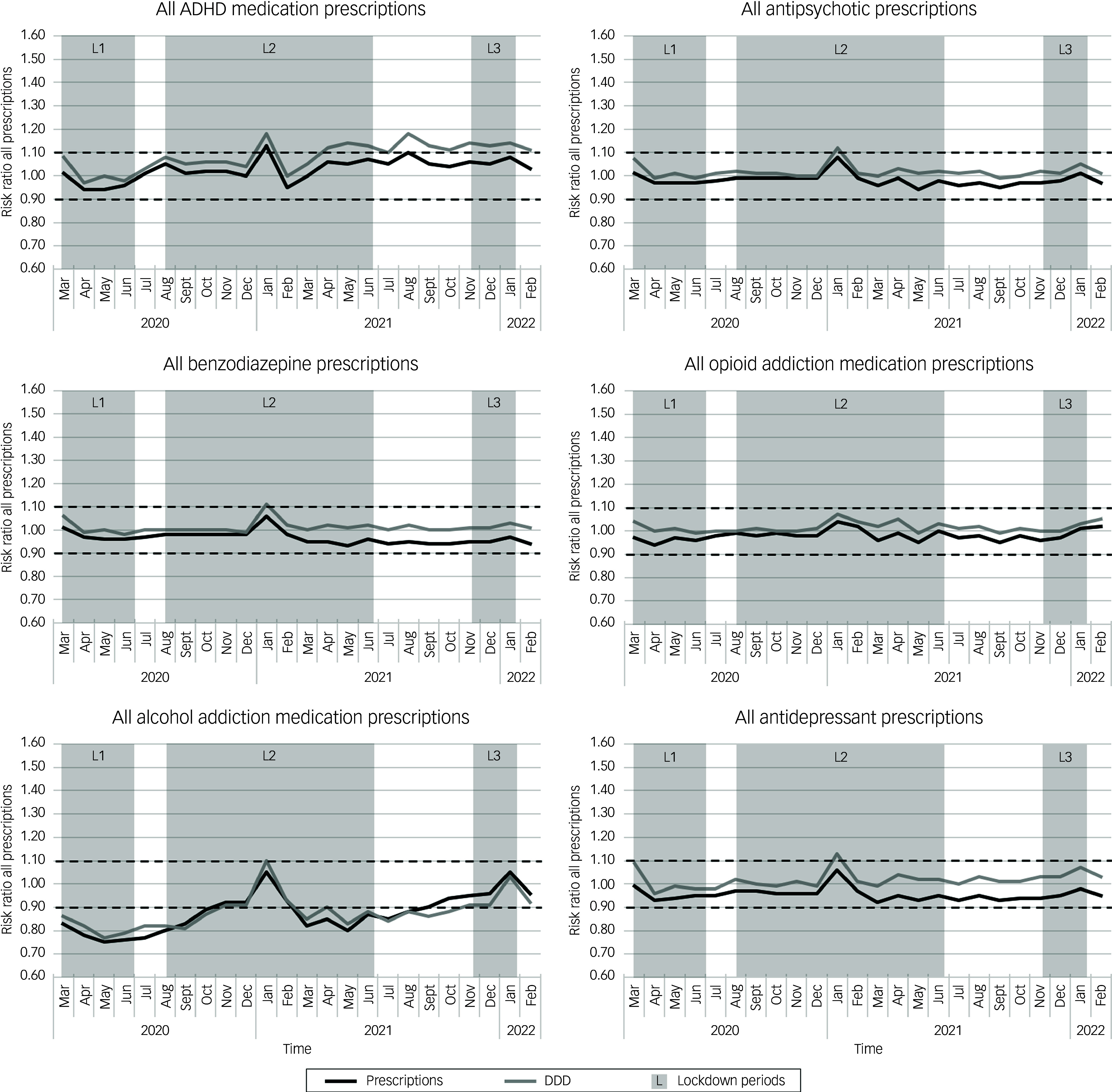
Lockdown-related dispensed prescription dynamics using monthly risk ratios for all dispensed prescriptions and defined daily doses (DDDs) in six psychotropic medication groups in The Netherlands. Risk ratios were calculated by comparing numbers of prescriptions or DDDs per 100 000 people per month in the COVID period with the same month in the reference year. Time periods indicated in grey depict the three lockdown periods, L1, L2 and L3 (Oxford COVID-19 Government Response Tracker^20^ score ≥ 50). Dashed lines represent risk ratios of ±0.1 above and below the baseline risk ratio of 1.0. Black lines indicate risk ratios for prescriptions; grey lines indicate risk ratios for DDD. ADHD, attention-deficit hyperactivity disorder.

**Fig. 3:**
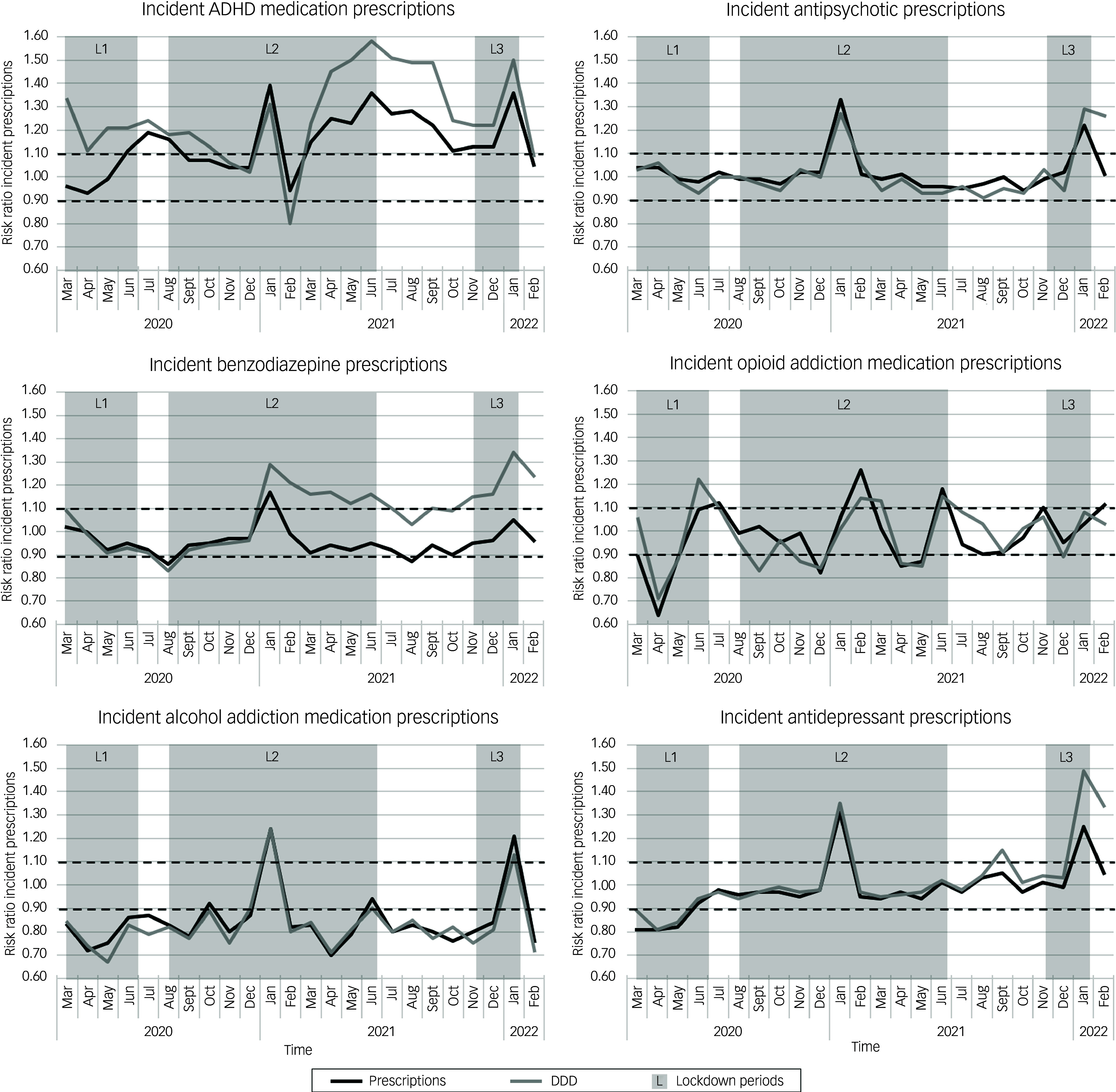
Lockdown-related dispensed prescription dynamics using monthly risk ratios of dispensed incident prescriptions and defined daily doses (DDDs) in six psychotropic medication groups in The Netherlands. Risk ratios were calculated by comparing numbers of prescriptions or DDDs per month in the COVID period with the same month in the reference year. Time periods indicated in grey depict the three lockdown periods, L1, L2 and L3 (Oxford COVID-19 Government Response Tracker^20^ score ≥ 50). Dashed lines represent risk ratios of ±0.1 above and below the baseline risk ratio of 1.0. Black lines indicate risk ratios for prescriptions; grey lines indicate risk ratios for DDD. ADHD, attention-deficit hyperactivity disorder.

For monthly lockdown-related risk ratio dynamics of all dispensed prescriptions, we observed a spike in monthly risk ratio in all medication groups for both number of total prescriptions and DDDs during the second lockdown in January 2021 (Fig. 2). For total DDDs, risk ratios exceeded the 1.1 threshold in all dispensed medication groups, except for all dispensed opioid addiction medication prescriptions, for which the risk ratio remained below the cut-off. Similar spikes in monthly risk ratios above the 1.1 cut-off value were observed for numbers of dispensed incident prescriptions and DDDs during the second and third lockdowns (Fig. 3 and Supplementary Fig. 4). Furthermore, the monthly risk ratio dynamics over time were more pronounced for incident psychotropic prescriptions compared with total prescription groups. See also Supplementary Table 2 for an overview of the average risk ratios in each phase of the pandemic.

For lockdown-related dynamics of dispensed ADHD medication prescriptions specifically, there was a persistent increase in the risk ratio for total prescription DDDs during 2021, with an average risk ratio of 1.12. For incident ADHD prescription DDDs, there was a relative increase in risk ratio during the first lockdown, with an average value of 1.21. Risk ratios for incident ADHD prescriptions also increased between the first and second lockdowns, reaching 1.19 in July 2020. Subsequently, there was an 11-month period starting in March 2021 during the second lockdown in which both DDDs and incident prescription numbers remained elevated above the 1.1 risk ratio cut-off (with average risk ratios of 1.23 for number of incident prescriptions and 1.40 for incident DDDs). Notably, during this period, there was a discrepancy in the risk ratios for dispensed prescriptions and dispensed DDDs, with DDD risk ratios remaining higher than those for dispensed prescriptions.

Monthly risk ratio dynamics for total and incident antipsychotic medication remained stable throughout the study period, with the exception of the aforementioned spikes in January of 2021 and 2022, which were mostly seen in the dispensed incident antipsychotic prescriptions group.

Lockdown-related monthly risk ratio dynamics also remained stable for total benzodiazepine prescriptions and total prescription DDDs. For the incident benzodiazepine prescriptions and DDDs, transient risk ratios of 0.86 and 0.83, respectively, were observed between the first and second lockdowns. This was followed by a sharp increase in dispensed incident prescription DDDs starting in January 2021 in the second lockdown period. This increase lasted throughout the rest of the study period, briefly dipping below the cut-off in August 2021 between the second and third lockdowns, with an average risk ratio between January 2021 and February 2022 of 1.17. Again, there was a discrepancy between the risk ratios in this timeframe for dispensed benzodiazepine prescriptions (average risk ratio: 0.96) compared with dispensed DDDs (average risk ratio: 1.17).

Total opioid addiction medication monthly risk ratio dynamics also remained stable during the study period and did not display the previously described spikes in January 2021 and 2022. However, monthly risk ratios for dispensed incident opioid addiction medication fluctuated above and below the cut-off range, mainly during the lockdown periods. The first lockdown period had the sharpest relative decline in incident dispensed prescriptions and DDDs, with risk ratios of 0.64 and 0.71, respectively, in April 2020. This was followed by a risk ratio peak for DDDs of 1.22 in June 2020. After this period, the monthly risk ratio dynamics for incident opioid addiction medication stabilised.

For total numbers of alcohol addiction medication prescription fills and DDDs, risk ratios were below the 0.9 cut-off between March and October 2020 and between March and August 2021. The average risk ratio throughout the whole COVID period was 0.88 for both total alcohol addiction prescriptions and DDDs. In addition to these spikes in numbers of incident prescriptions and DDDs for alcohol addiction medication, there was an overall decreased average risk ratio for incident prescriptions and DDDs during the entire COVID period, with average risk ratios of 0.85 for dispensed prescriptions and 0.83 for DDDs.

Total antidepressant monthly risk ratio prescribing dynamics remained stable, excluding a transient DDD risk ratio of 1.13 in January of 2021. However, in incident antidepressant prescription risk ratios, there was a notable decrease in the relative number of prescriptions during the first lockdown, with an average risk ratio of 0.84 in this period. This corresponded with approximately 15 000 fewer incident prescriptions compared with the pre-COVID reference period. There was also a brief period between the second and third lockdown periods in September of 2021 when the risk ratio of dispensed DDDs increased to 1.15. During the third lockdown, there was again a sharp rise in risk ratios for numbers and DDDs of incident antidepressants, with DDD risk ratios remaining elevated at 1.34 during February 2022.

## Discussion

This study describes the changes in trends and dynamics of dispensed psychotropic prescriptions in The Netherlands during the COVID-19 pandemic and associated lockdown periods. No overall pandemic-related changes in dispensed prescription trends were found, except for alcohol addiction medication, for which the pre-pandemic declining trend levelled off during the pandemic. Notable monthly dispensed prescription dynamics were observed in all medication groups, mostly during lockdown periods. Most of the individual medication groups displayed overlapping lockdown-related dynamics, such as transient increases in the relative amounts of dispensed prescriptions and DDDs in January during the second and third lockdowns. However, there were medication-group-specific lockdown-related dynamics for dispensed (incident) ADHD medication, benzodiazepine, opioid addiction medication and antidepressant prescriptions. These findings suggest a potentially relevant impact of mainly lockdown-related effects on Dutch prescribing practices of psychotropic medications during the COVID-19 pandemic. Possible drivers of these changes and their implications will be discussed further.

A recurrent pattern seen across most psychotropic medication groups was a spike in relative amounts of dispensed prescriptions and DDDs during the second and third lockdown periods. This corresponded with a relative increase in dispensed prescriptions and DDDs during the first week of January. In The Netherlands, more prescriptions are usually filled in the last weeks of the year^19^ and fewer in the first week. This is due to the reset of the mandatory deductible for medical treatment at the change of each calendar year. It could therefore be hypothesised that more patients retrieved their psychotropic medication prescriptions during the first weeks of January owing to the high government response score and uncertainty during this period (Supplementary Fig. 2). At this time, face-to-face visits to doctors were discouraged; thus, it is conceivable that physicians were more liberal in prescribing (incident) psychiatric medications. Alternatively, the finding could be explained by an increase in psychiatric symptoms around this time, again potentially driven by lockdown-related factors such as curfews and school closures. It remains uncertain whether this phenomenon is unique to psychotropic medications or whether a similar pattern could be seen across multiple somatic medication prescriptions in The Netherlands.

In the analysis of individual psychotropic medication groups, ADHD medication prescription dynamics showed a sharp rise in incident prescriptions and DDDs in 2021. This was in line with the findings of a recent meta-analysis describing a global increase in ADHD symptoms during the pandemic,^21^ as well as a large US study^6^ that detected a sharp increase in incident ADHD prescriptions. Furthermore, another international study found that initial decreases in global ADHD medication DDDs in 2020 were followed by increases in DDDs in 2021.^22^ A plausible driver of these findings is that during lockdowns, family members were more aware of each other’s symptoms and therefore (re)initiated pharmacotherapy.^23^ Also, frequent video-calling for work or school may have driven individuals with ADHD to (re)initiate medicinal treatment of their ADHD symptoms to improve concentration.^24^ The discrepancy between the dispensed incident prescription risk ratio and incident DDD risk ratio could have been indicative of these new patients receiving larger drug volumes in this time frame. It is therefore recommended to monitor patients who (re)initiated their medication during COVID to investigate whether continuation of treatment is still warranted.

Antipsychotic medication prescribing in general displayed no notable lockdown-related dynamics throughout the pandemic, for either all or incident dispensed prescriptions. This could be indicative of adequate continuity and accessibility of care; however, there is limited (inter)national evidence regarding changes in incidence and prevalence of psychotic disorders during the pandemic.^5^ The findings of this study are also in line with those of an English study^13^ reporting that antipsychotic prescribing remained unchanged during the pandemic. French^8^ and Canadian^9^ studies reported slight increases in dispensed antipsychotic prescriptions during the pandemic. The increase found in Canada was partially driven by off-label prescribing to nursing home residents and patients with dementia;^9^ this was also found in a different English study.^25^

During the second lockdown, DDDs of dispensed incident benzodiazepine prescriptions increased notably and remained elevated for the rest of the pandemic when compared with the reference year. However, dispensed incident benzodiazepine prescription numbers declined on average during this period. These results, taken together, indicate that incident benzodiazepine prescriptions became larger from the beginning of 2021, which was during the most severe government COVID response, including a national curfew. It could therefore be hypothesised that new benzodiazepine recipients in The Netherlands acquired larger first prescriptions to ‘bridge’ a lockdown period and limit pharmacy visits.^26^ Patients exposed to larger amounts of first benzodiazepine prescriptions can be at elevated risk of dependence in the long term.^27^ Therefore, patients who received a new benzodiazepine prescription during the COVID-19 period should also be monitored closely.

Total dispensed opioid addiction medication prescriptions remained notably stable during the pandemic, with this medication group not displaying the aforementioned transient spikes in January 2021 and 2022. Most of these prescriptions were repeat prescriptions, with only 1% being incident prescriptions. It seems therefore that the COVID-19 pandemic had a limited impact on continuity of care for existing patients receiving opioid addiction medication. However, incident opioid addiction medication prescriptions fluctuated strongly, with most peaks and dips occurring during the first and second lockdowns. The sizable drop in prescriptions in April and May 2020 corresponded with recent Dutch research that found a decline in incident opioid prescriptions during the first lockdown.^18^ Suspected drivers of overall opioid prescribing are thought to be the decline in elective surgeries and fewer injuries during lockdowns. In the case of incident opioid addiction medication, the decline in the first lockdown is suspected to have been due to reduced accessibility of opioid addiction care. The average numbers of incident opioid addiction medication prescriptions during the whole pandemic remained similar to those of the reference year, suggesting that the decline in the first lockdown was later compensated for.

The only medication group in which a significant overall pandemic-related change in dispensed prescription trends could be detected was the total alcohol addiction medication group. The pre-pandemic decline in total alcohol addiction medication prescriptions and DDDs halted at the onset of the pandemic, as shown in the ITSA. However, no such overall pandemic-related trend change was seen for dispensed incident alcohol addiction prescriptions. This was in contrast to a Dutch^28^ study showing a significant decrease in the 6-month prevalence of alcohol use disorders during the pandemic. The lockdown-related monthly dynamics for dispensed incident prescriptions decreased on average throughout the pandemic; this could be explained by a pre-pandemic declining trend in dispensed incident prescriptions. These results combined indicate that existing users adhered more to their medication during the pandemic than before the pandemic. Other Dutch national healthcare data showed that healthcare professionals provided more weekly contact hours to patients with alcohol-related disorders during the pandemic compared with before,^29^ potentially stimulating pharmacotherapeutic adherence.^30^ It is also possible that patients had fewer relapses owing to social restrictions that limited the exposure of the general public to alcohol;^31^ this, in turn, could have stimulated users to continue pharmacological treatment.

Finally, in the antidepressant medication group, lockdown-related monthly risk ratio dynamics showed decreases in dispensed incident antidepressant prescriptions and DDDs during the first lockdown; however, total dispensed antidepressant prescriptions remained stable. This corresponded with Canadian^9^ and US^6,32^ findings of a decline in incident antidepressant prescriptions during the first public health measures. However, Scandinavian^7^ and English^12^ researchers have reported increases in total antidepressant medication prescriptions at the pandemic’s onset. The results of this study could be indicative of new patients with depressive symptoms experiencing difficulties or hesitancy in accessing their general practitioner or mental health services during the first lockdown.^33^ Physicians may also have been less inclined to initiate or switch antidepressant treatment while the healthcare system was adjusting to the pandemic and in-person visits were discouraged.^34^ Therefore, a significant number of individuals may have abstained from antidepressant treatment at the start of the pandemic. Subsequently, a greater increase in dispensed DDDs of incident antidepressant prescriptions compared with dispensed prescriptions was seen from January 2022 onwards. This was probably due to an increase in the average size of incident antidepressant prescriptions during the third lockdown period, which could be indicative of stockpiling rather than an increase in the incidence of depression within the population.

### Strengths and limitations

This study describes overall pandemic-related changes in dispensed prescription trends and lockdown-related monthly dispensed prescription dynamics in the broadest set of psychotropic medications during the COVID-19 pandemic to date, covering 96% of the entire Dutch out-patient population. A limited number of studies have also separately investigated incident and total prescriptions.^9,18^ The inclusion of dispensed DDDs by medication group in our study provided more insight with respect to changes in amounts of prescribed medications during the pandemic. Furthermore, the use of time-series prescription data expressed as monthly risk ratios (adjusted for population growth) provided a high resolution view of the relative monthly prescribing dynamics. This made it possible for us to detect potential lockdown-related changes in psychiatric health(care) throughout the pandemic and simultaneously correct for seasonal prescribing fluctuations. Finally, all of the requested ATC codes require a doctor’s prescription in The Netherlands; this supports the use of these data as a proxy for healthcare provision.

Regarding the limitations of this study, only aggregated prescription data could be used owing to the privacy policy of the national registry. This limited the scope of statistical methods that could be applied. In addition, it was not possible to distinguish pandemic-related changes from seasonal fluctuations in the ITSA, owing to the reference period being 1 year. Furthermore, there can be discrepancies between dispensed medications versus prescribed medications due to several factors, including primary nonadherence to pharmacotherapy. However, a previous Dutch study found that approximately 7% of prescribed psychiatric medications were not collected by patients in The Netherlands. Thus, we believe the difference between prescribed medication and dispensed medication in this study was modest.^35^ Next, the database did not contain data on indications for the dispensed prescriptions analysed; this limited our ability to correlate the findings with changes in the incidence and prevalence of psychiatric disorders. Finally, the observational and explorative nature of the study also hindered any definite causal inferences regarding the findings.

### Implications and future directions

No overall changes in dispensed prescription trends of psychotropic medications were found during the pandemic, except for all alcohol addiction medication prescriptions. Lockdown-related monthly dispensed prescription dynamics were mostly observed for incident prescription numbers and DDDs and during different lockdown periods. This was most notable for (incident) ADHD medication, incident benzodiazepines, incident opioid addiction medication and incident antidepressant prescriptions. These findings are indicative of possible over- or under-prescribing within the aforementioned medication groups during the pandemic in The Netherlands. Clinicians should therefore be vigilant regarding potential unwarranted enduring effects of the pandemic on prescriptions of psychotropic medication for their patients. Further research is needed to identify and clarify the specific (inter)national drivers of the observed changes in prescribing rates, such as the accessibility and quality of healthcare during crises. This knowledge will help us to anticipate future mental health(care) challenges.

## Supporting information

Visser et al. supplementary material 1 Visser et al. supplementary material

Visser et al. supplementary material 2 Visser et al. supplementary material

Visser et al. supplementary material 3 Visser et al. supplementary material

Visser et al. supplementary material 4 Visser et al. supplementary material

Visser et al. supplementary material 5 Visser et al. supplementary material

Visser et al. supplementary material 6 Visser et al. supplementary material

## Data Availability

The data that support the study findings and the analytic code used in the study will be available in the Radboud Data Repository at https://data.ru.nl/ upon publication or directly from the corresponding author, D.A.V., on reasonable request.
