# Supplementary material for: Fluctuations in dispensed out-patient psychotropic medication prescriptions during the COVID-19 pandemic in The Netherlands
Source: BJPsych Open. 2025 Mar 20;11(2):e64. doi: 10.1192/bjo.2024.867 (PMC12001946; doi:10.1192/bjo.2024.867)
Supplement: Visser et al. supplementary material 1 — Visser et al. supplementary material [file S2056472424008676sup001.docx]

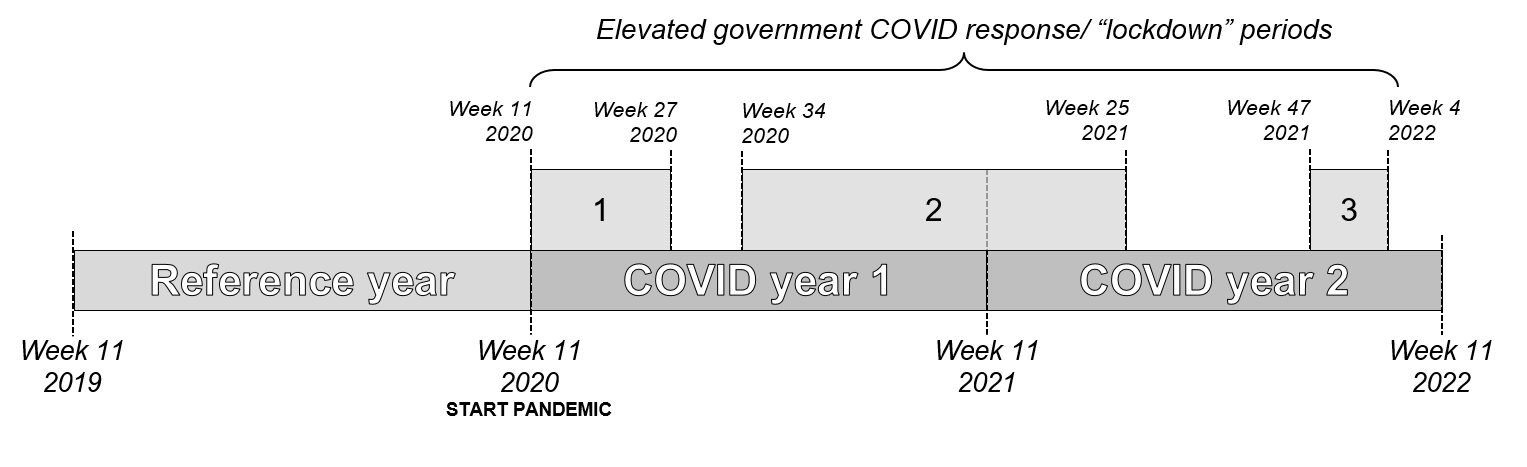


**Supplementary Fig. 1** Graphical overview of the time periods utilised in this study: a single reference year (week 11 of 2019 to week 10 of 2020) together with two years during the COVID-19 pandemic (week 11 of 2020 to week 10 of 2022). Three lockdown periods are annotated above the COVID period.
