# Supplementary material for: Fluctuations in dispensed out-patient psychotropic medication prescriptions during the COVID-19 pandemic in The Netherlands
Source: BJPsych Open. 2025 Mar 20;11(2):e64. doi: 10.1192/bjo.2024.867 (PMC12001946; doi:10.1192/bjo.2024.867)
Supplement: Visser et al. supplementary material 2 — Visser et al. supplementary material [file S2056472424008676sup002.docx]

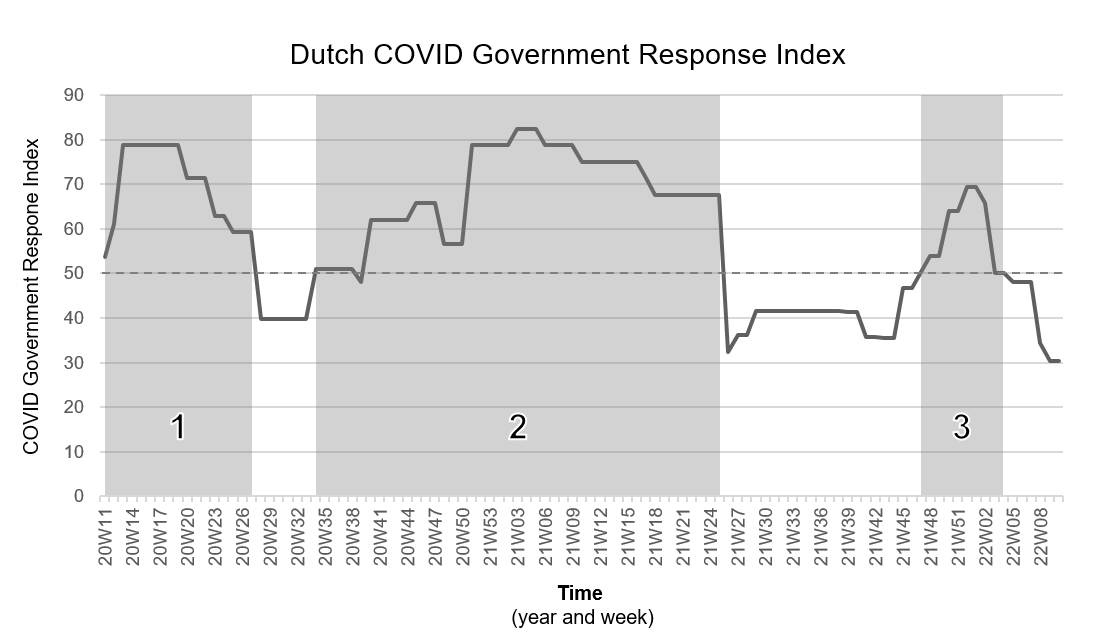


**Supplementary Fig. 2** Dutch COVID Government Response Index over time. Based on the Oxford COVID-19 Government Response Tracker (OxCGRT) data from Hale et al., 2021. This is a longitudinal global panel database of pandemic policies, numerically expressed as an aggregated value based on nine policy indicators (e.g. workplace/school closures). Data were extracted from the database as a continuous scale from 0 (least strict response) to 100 (strictest response). The weighted average stringency index for vaccinated and non-vaccinated individuals was used from September 25th 2021 onwards. Time periods indicated in grey depict the three periods of elevated COVID government response in the Netherlands referred to as “lockdowns” in this study. The grey dotted line indicates the cut-off score of 50 used in this study to determine “periods of elevated COVID government response”. There was a one-week period in the second EGCR period where the score was <50, however since this period was less than 4 weeks it was incorporated in the second EGCR period.
