# Supplementary material for: Fluctuations in dispensed out-patient psychotropic medication prescriptions during the COVID-19 pandemic in The Netherlands
Source: BJPsych Open. 2025 Mar 20;11(2):e64. doi: 10.1192/bjo.2024.867 (PMC12001946; doi:10.1192/bjo.2024.867)
Supplement: Visser et al. supplementary material 4 — Visser et al. supplementary material [file S2056472424008676sup004.docx]

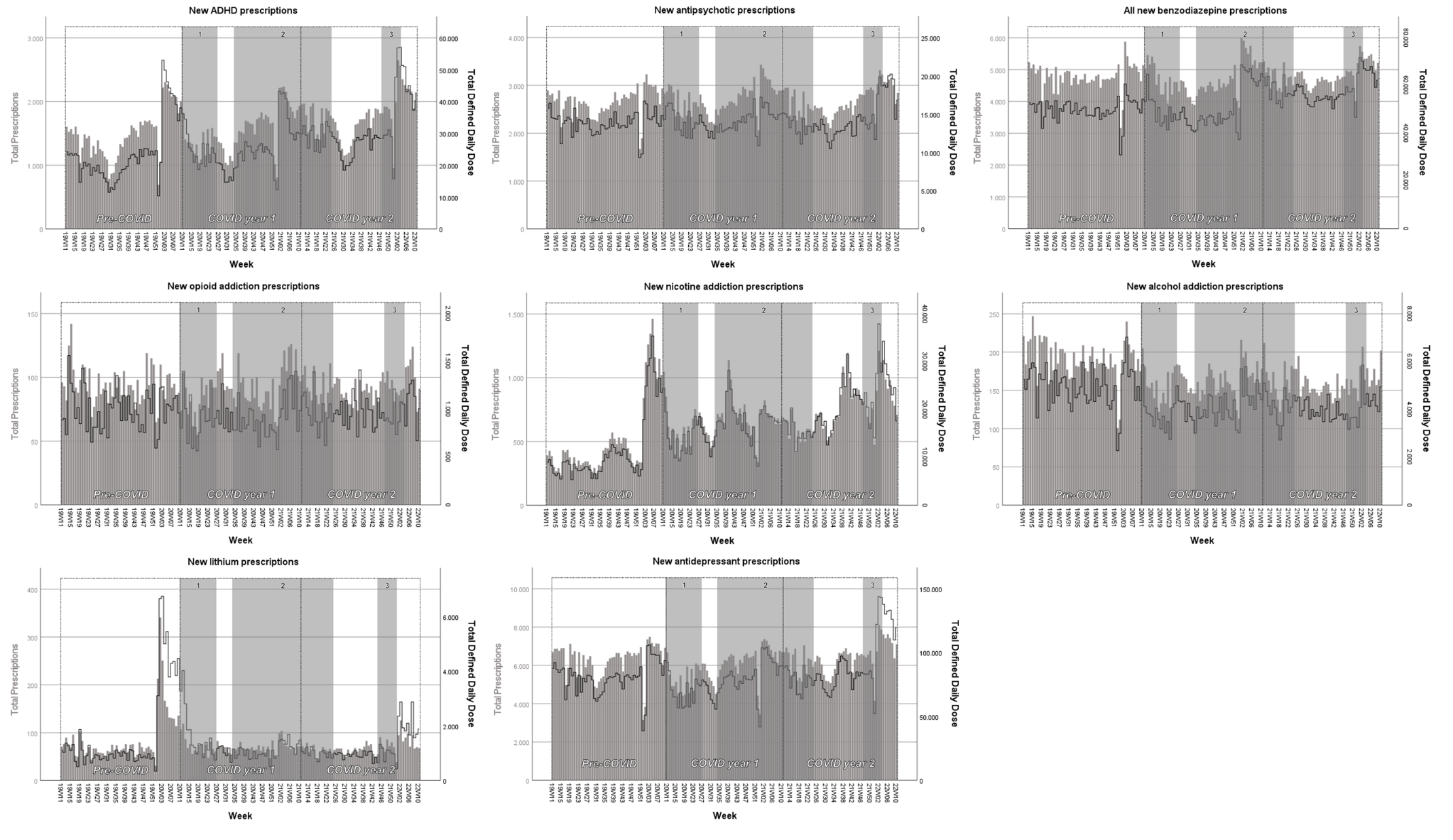


**Supplementary Fig. 4** Absolute *incident* prescriptions per calendar week (grey bars) and defined daily doses (black lines) per main drug group over the course of three years (one reference year and two COVID years). Time periods indicated in grey depict the three lockdown periods. “Nicotine addiction” and “lithium” groups are also included in this figure.
