# Supplementary material for: Fluctuations in dispensed out-patient psychotropic medication prescriptions during the COVID-19 pandemic in The Netherlands
Source: BJPsych Open. 2025 Mar 20;11(2):e64. doi: 10.1192/bjo.2024.867 (PMC12001946; doi:10.1192/bjo.2024.867)
Supplement: Visser et al. supplementary material 5 — Visser et al. supplementary material [file S2056472424008676sup005.docx]

| **Supplementary Table 1** List of medication groups and corresponding ATC codes including their percentual representation of the total number of prescriptions in the medication group (listed in descending order per medication group). ATC codes requested from the GIP with no corresponding data are listed in the bottom row of the table. | | | |
| --- | --- | --- | --- |
| **Medication group** (% prescriptions of main group) | **ATC group code** | **ATC** (n) | **Individual ATC**  (ATC code, % of prescriptions in subgroup), sorted by %, descending |
| **ADHD Medication** | *N06BA-* | **4** | methylphenidate (N06BA04, 79%), dexamphetamine (N06BA02, 15%),  lisdexamphetamine (N06BA12, 4%), atomoxetine (N06BA09, 2%) |
| **Antipsychotics** | *N05A-* | **23** | - |
| *Typical antipsychotics (16)* | *N05A(C/D/F/G)-* | *10* | haloperidol (N05AD01, 45%), pipamperone (N05AD05, 17%), zuclopenthixol (N05AF05, 11%),  pimozide (N05AG02, 8%), penfluridol (N05AG03, 8%), flupentixol (N05AF01, 7%),  chlorprothixene (N05AF03, 4%), periciazine (N05AC01, 1%), bromperiodol (N05AD06, 0%),  fluspirilene (N05AG01, 0%) |
| *Atypical antipsychotics (84)* | *N05A(X/H/E/L)-* | *13* | quetiapine (N05AH04, 43%), olanzapine (N05AH03, 20%), risperidone (N05AX08, 14%),  clozapine (N05AH02, 12%), aripiprazole (N05AX12, 9%), paliperidone (N05AX13, 1%),  sulpiride (N05AL01, 1%), amisulpride (N05AL05, 0%), lurasidone (N05AE05, 0%),  brexpiprazole (N05AX16, 0%), cariprazine (N05AX15, 0%), sertindole (N05AE03, 0%),  tiapride (N05AL03, 0%) |
| **Benzodiazepines** | *N05(B/C)-* | **17** | oxazepam (N05BA04, 25%), temazepam (N05CD07, 18%), lorazepam (N05BA06, 15%),  diazepam (N05BA01, 12%), zopiclone (N05CF01, 7%), zolpidem (N05CF02, 6%),  alprazolam (N05BA12, 6%), midazolam (N05CD08, 5%), lormetazepam (N05CD06, 2%),  clorazepate (N05BA05, 2%), bromazepam (N05BA08, 1%), flurazepam (N05CD01, 1%),  flunitrazepam (N05CD03, 0%), brotizolam (N05CD09, 0%), loprazolam (N05CD11, 0%),  prazepam (N05BA11, 0%), nitrazepam (N05CD02, 0%) |
| **Opioid addiction** | *N07BC-* | ***3*** | methadone (N07BC02, 92%), buprenorphine/naloxone (N07BC51, 8%), buprenorphine (N07BC01, 0%) |
| **Alcohol addiction** | *N07BB-* | ***4*** | disulfiram (N07BB01, 50%), naltrexone (N07BB04, 27%), acamprosate (N07BB03, 19%),  nalmefene (N07BB05, 4%) |
| **Antidepressants** | *N06A-* | **22** | - |
| *SSRI (48)* | *N06AB-* | *6* | citalopram (N06AB04, 34%), paroxetine (N06AB05, 23%), sertraline (N06AB06, 17%),  escitalopram (N06AB10, 13%), fluoxetine (N06AB03, 9%), fluvoxamine (N06AB08, 3%) |
| *TCA (24)* | *N06AA-* | *6* | amitryptyline (N06AA09, 55%), nortriptyline (N06AA10, 31%), clomipramine (N06AA04, 13%),  imipramine (N06AA02, 1%), maprotiline (N06AA21, 0%), doxepin (N06AA12, 0%) |
| *SNRI (13)* | *N06AX-* | *2* | venlafaxine (N06AX16, 79%), duloxetine (N06AX21, 21%) |
| *MAOi (0)* | *N06A(G/F)-* | *3* | tranylcypromine (N06AF04, 76%), moclobemide (N06AG02, 21%), phenelzine (N06AF03, 3%) |
| *Other antidepressants (15)* | *N06AX-* | *5* | mirtazapine (N06AX11, 74%), bupropion (N06AX12, 14%), trazodone (N06AX05, 9%),  vortioxetine (N06AX26, 3%), mianserin (N06AX03, 0%) |
| ***Not retrieved from GIP database*** | *N/A* | *7* | *Ephedrine combinations (A08AA56), dapoxetine (G04BX14), oxycodone with naltrexone (N02AA56), methadone in combination with non-neuroleptics (N02AC52), lorazepam combinations (N05BA56), amitriptyline and psycholeptics (N06CA01) and fluoxetine and psycholeptics (N06CA03).* |
| ***Retrieved from GIP database, but excluded from study*** | *N07BA- and*  *N05AN01* | *3* | *nicotine (N07BA01), varenicline (N07BA03), lithium (N05AN01)* |
