# Supplementary material for: Fluctuations in dispensed out-patient psychotropic medication prescriptions during the COVID-19 pandemic in The Netherlands
Source: BJPsych Open. 2025 Mar 20;11(2):e64. doi: 10.1192/bjo.2024.867 (PMC12001946; doi:10.1192/bjo.2024.867)
Supplement: Visser et al. supplementary material 6 — Visser et al. supplementary material [file S2056472424008676sup006.docx]

| **Supplementary Table 2a** Average risk ratios for Rx/DDDs per stage of the COVID-19 pandemic in the Netherlands. | | | | | | | | | |
| --- | --- | --- | --- | --- | --- | --- | --- | --- | --- |
| **Periods →**  **↓Medication groups** | **Lockdown 1**  (Rx/DDD) | **Interim period 1**  (Rx/DDD) | | **Lockdown 2**  (Rx/DDD) | | **Interim period 2**  (Rx/DDD) | **Lockdown 3**  (Rx/DDD) | | **Interim period 3**  (Rx/DDD) |
| *All prescriptions* |  |  | |  | |  |  | |  |
| ADHD medication | 0.96/1.01 | 1.03/1.06 | | 1.03/1.08 | | 1.06/**1.13*** | 1.07/**1.14*** | | 1.03/**1.11*** |
| Antipsychotics | 0.98/1.02 | 0.99/1.02 | | 0.99/1.02 | | 0.96/1.01 | 1.00/1.03 | | 0.97/1.01 |
| Benzodiazepines | 0.98/1.01 | 0.98/1.00 | | 0.98/1.02 | | 0.94/1.01 | 0.96/1.02 | | 0.94/1.01 |
| Opioid addiction medication | 0.96/1.01 | 0.99/1.00 | | 0.99/1.02 | | 0.97/1.01 | 0.99/1.02 | | 1.02/1.05 |
| Alcohol addiction medication | **0.78***/**0.81*** | **0.79***/**0.82*** | | **0.88***/**0.89*** | | **0.90***/**0.87*** | 1.01/0.97 | | 0.96/0.92 |
| Antidepressants | 0.95/1.01 | 0.96/1.00 | | 0.96/1.02 | | 0.94/1.02 | 0.97/1.05 | | 0.95/1.03 |
| *Incident prescriptions* |  |  | |  | |  |  | |  |
| ADHD medication | 1.00/**1.22*** | **1.18***/**1.21*** | | **1.15***/**1.22*** | | **1.20***/**1.39*** | **1.25***/**1.36*** | | 1.05/1.09 |
| Antipsychotics | 1.01/1.00 | 1.01/1.00 | | 1.02/1.00 | | 0.97/0.96 | **1.12***/**1.12*** | | 1.01/**1.26*** |
| Benzodiazepines | 0.97/0.98 | **0.89***/**0.87*** | | 0.96/1.06 | | 0.92/1.09 | 1.01/**1.25*** | | 0.96/**1.24*** |
| Opioid addiction medication | **0.88***/0.97 | 1.06/1.03 | | 1.00/0.96 | | 0.96/1.02 | 0.99/0.99 | | **1.11***/1.03 |
| Alcohol addiction medication | **0.79***/**0.77*** | **0.85***/**0.81*** | | **0.87*0.86*** | | **0.80***/**0.80*** | 1.03/0.97 | | **0.76***/**0.72*** |
| Antidepressants | **0.84***/**0.87*** | 0.97/0.96 | | 1.00/1.01 | | 1.01/1.04 | **1.12***/**1.26*** | | 1.05/**1.34*** |
| **Supplementary Table 2b** Average risk ratios for Rx/DDDs per phase of the COVID-19 pandemic (lockdowns, interim periods and during the whole pandemic) in the Netherlands. | | | | | | | | | |
| **Periods →**  **↓Medication groups** | | | **All lockdowns**  (Rx/DDD) | | **Interim periods**  (Rx/DDD) | | | **Whole pandemic**  (Rx/DDD) | |
| *All prescriptions* | | |  | |  | | |  | |
| ADHD medication | | | 1.02/1.07 | | 1.05/**1.11*** | | | 1.03/1.08 | |
| Antipsychotics | | | 0.99/1.02 | | 0.97/1.01 | | | 0.98/1.02 | |
| Benzodiazepines | | | 0.97/1.01 | | 0.95/1.01 | | | 0.97/1.01 | |
| Opioid addiction medication | | | 0.98/1.02 | | 0.98/1.01 | | | 0.98/1.02 | |
| Alcohol addiction medication | | | **0.87***/**0.88*** | | **0.88***/**0.87*** | | | **0.88***/**0.88*** | |
| Antidepressants | | | 0.96/1.02 | | 0.95/1.01 | | | 0.96/1.02 | |
| *Incident prescriptions* | | |  | |  | | |  | |
| ADHD medication | | | **1.13***/**1.24*** | | **1.18***/**1.31*** | | | **1.14***/**1.26*** | |
| Antipsychotics | | | 1.03/1.02 | | 0.98/1.01 | | | 1.02/1.01 | |
| Benzodiazepines | | | 0.97/1.07 | | 0.92/1.06 | | | 0.96/1.07 | |
| Opioid addiction medication | | | 0.97/0.97 | | 1.01/1.02 | | | 0.98/0.99 | |
| Alcohol addiction medication | | | **0.87***/**0.85*** | | **0.81***/**0.79*** | | | **0.85***/**0.83*** | |
| Antidepressants | | | 0.98/1.00 | | 1.00/1.06 | | | 0.98/1.03 | |
| Abbreviations: DDD, Defined daily dose; ADHD, attention deficit hyperactivity disorder; Rx, prescriptions.  Lockdown period 1: week 11 of 2020 to week 27 of 2020. Lockdown period 2: week 34 of 2020 to week 25 of 2021.  Lockdown period 3: week 47 of 2021 to week 4 of 2022.  RRs were calculated per month by dividing the number of prescriptions (or DDDs) per 100.000 inhabitants during the COVID month by the number of prescriptions (or DDDs) per 100.000 inhabitants of the same month in the reference period. Average RRs were calculated by taking the average RR within a given lockdown/interim period.  *****=RR ±0.10 above or below a zero-change RR of 1.00. | | | | | | | | | |
